# Voltage sensitive phosphatases: emerging kinship to protein tyrosine phosphatases from structure-function research

**DOI:** 10.3389/fphar.2015.00020

**Published:** 2015-01-10

**Authors:** Kirstin Hobiger, Thomas Friedrich

**Affiliations:** ^1^Department of Neurophysiology, Institute of Physiology and Pathophysiology, Philipps-Universität MarburgMarburg, Germany; ^2^Max-Volmer-Laboratory of Biophysical Chemistry, Institute of Chemistry, Technische Universität BerlinBerlin, Germany

**Keywords:** cysteine-based phosphatase, dual-specific phosphatase, acid phosphatase, low molecular weight phosphatase, Cdc25, phosphoinositide, phosphotyrosine, phosphoproteins

## Abstract

The transmembrane protein Ci-VSP from the ascidian *Ciona intestinalis* was described as first member of a fascinating family of enzymes, the voltage sensitive phosphatases (VSPs). Ci-VSP and its voltage-activated homologs from other species are stimulated by positive membrane potentials and dephosphorylate the head groups of negatively charged phosphoinositide phosphates (PIPs). In doing so, VSPs act as control centers at the cytosolic membrane surface, because they intervene in signaling cascades that are mediated by PIP lipids. The characteristic motif CX_5_RT/S in the active site classifies VSPs as members of the huge family of cysteine-based protein tyrosine phosphatases (PTPs). Although PTPs have already been well-characterized regarding both, structure and function, their relationship to VSPs has drawn only limited attention so far. Therefore, the intention of this review is to give a short overview about the extensive knowledge about PTPs in relation to the facts known about VSPs. Here, we concentrate on the structural features of the catalytic domain which are similar between both classes of phosphatases and their consequences for the enzymatic function. By discussing results obtained from crystal structures, molecular dynamics simulations, and mutagenesis studies, a possible mechanism for the catalytic cycle of VSPs is presented based on that one proposed for PTPs. In this way, we want to link the knowledge about the catalytic activity of VSPs and PTPs.

## INTRODUCTION

Phosphorylation and dephosphorylation of cellular substrates, such as proteins, lipids, carbohydrates or nucleic acids, are crucial for the precise spatiotemporal transduction of signals across the cellular space. While phosphorylation of substrates is mediated by kinases, phosphatases attack their substrates by hydrolyzing covalently attached phosphate groups. In this context, protein tyrosine phosphatases (PTPs^1^) come into play with their dephosphorylation activity toward a diverse pool of cellular substrates. Members of this huge protein family are involved in multiple processes like endo- and exocytosis, cell differentiation, cell proliferation and migration – to list only a few examples. Because of this broad spectrum in activity, it is not surprising that malfunctions of PTPs are associated with various diseases, for example neurological disorders, diabetes, or cancer (Cohen, 2001; Tautz et al., 2013).

Most PTPs are very similar in the structure of their catalytic domain. Consequently, a medicinal treatment that should be specific just for one phosphatase bears always the risk of unwanted side effects since the drug may act on more than one of these enzymes. Therefore, the development of new medications requires a detailed knowledge about the individuality of PTPs, and here – in particular – about the molecular details of substrate recognition, activation and catalysis, about the mechanisms that regulate their activity, and about their functional role in specific signaling cascades (Zhang, 2001; Scott et al., 2010; He et al., 2014).

Voltage-sensitive phosphatases (VSPs) belong to the PTP family due to their homology in amino acid sequence of the active site and similarities in their structure of the catalytic domain. In VSPs, the phosphatase activity is directly coupled to the membrane potential. This voltage-switchable activity qualifies VSPs as ideal model systems to study enzymatic mechanisms of PTPs under defined experimental conditions.

Since the first identification of a PTP in Tonks et al. (1988a,b), a lot of studies have been performed leading to a huge amount of detailed information about these enzymes. However, although VSPs are members of the PTP family, their relationship has only been marginally discussed so far. In this review, we will address structural and mechanistic similarities of VSPs and PTPs. By linking the knowledge about these phosphatases, we want to answer the question if the results achieved for VSPs can be integrated into the set of principles which have already been described in more detail for PTPs.

### GENERAL CLASSIFICATION OF PTPs

The classification of PTPs is primarily based on their catalytic mechanism. Two different kinds of PTP subfamilies exist, the so called cysteine-based and the aspartate-based PTPs. Here, the name implies the key residue that mediates the dephosphorylation step in the reaction cycle of the respective class of enzymes. Although using a similar pool of substrates, cysteine- and aspartate-based PTPs are completely different in structure and the enzymatic reaction mechanism. Because this review will focus on cysteine-based PTPs, the reader interested in aspartate-based PTPs is referred to the excellent reviews by Moorhead et al. (2007, 2009).

The hallmark of all cysteine-based PTPs is the CX_5_RS/T sequence motif in their active site. Besides this so-called PTP recognition loop or P-loop, cysteine-based PTPs can be further categorized into three subclasses, I to III, based on their similarities in amino acid sequence, structure, and substrate specificity. The excellent overview of Alonso et al. (2004) is recommended here for a detailed classification of cysteine-based PTPs. However, for the sake of completeness, a short summary about the three PTP subclasses is also given here.

#### Class I PTPs – the classical and dual-specific PTPs

**FIGURE 1:**
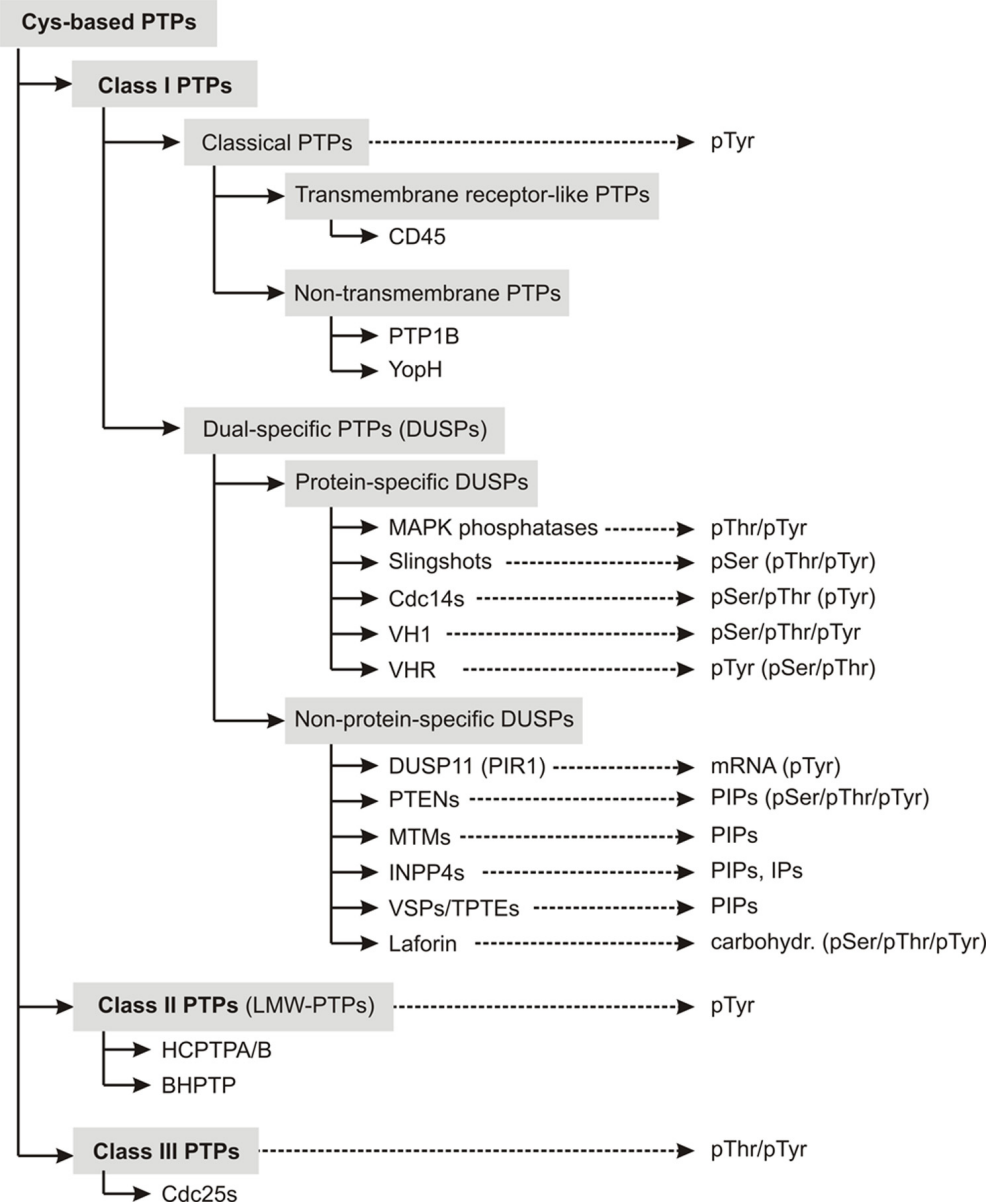
**Overview about the classification scheme of cysteine-based PTPs.** Members of individual subfamilies, which were exemplarily chosen and discussed in the main text, are listed in their respective PTP subclass. The preferred substrates of these phosphatases are given. Substrates that can be dephosphorylated, but with less preference are mentioned in parentheses. Abbreviations used here are explained in Table S1.

Class I PTPs form the largest subfamily of cysteine-based PTPs known so far. Their expression has been detected in all forms of life, from bacteria, yeast, plants to mammals (Pawson and Scott, 2005; Moorhead et al., 2009). This class of phosphatases comprises two subfamilies, the “classical” and the “dual-specific” PTPs (**Figure 1**).

***Classical PTPs*** show strict substrate specificity for phosphotyrosine (pTyr) residues. They are further categorized into receptor-like PTPs (rPTPs), which are anchored in the membrane, and non-transmembrane PTPs, which are located intracellularly.

A famous example for a receptor-like classical PTP is CD45, a transmembrane PTP that is expressed on the surface of leucocytes. Particularly in T lymphocytes, CD45 plays an essential role in the signal transduction that is initiated through the antigen-stimulation of the T cell receptor at the membrane surface (Altin and Sloan, 1997; Hermiston et al., 2003).

One prominent example for a non-transmembrane classical PTP is PTP1B, the founding member of the cysteine-based PTP superfamily (Tonks et al., 1988a,b). This soluble enzyme has attracted special attention in recent years, because it is well-known to act as negative regulator in the insulin signaling pathway. Therefore, it is a core subject of current pharmaceutical research as potential target for the treatment of type 2 diabetes and obesity (Goldstein, 2001; Zhang and Zhang, 2007; Combs, 2010). Besides that, PTP1B has also been discussed as putative drug target for breast cancer therapies (Yip et al., 2010; Feldhammer et al., 2013). However, interpretation of experimental results in this field needs careful consideration since there are hints that PTP1B might act in both directions, tumor suppression and promotion (Lessard et al., 2010).

In contrast to classical PTPs, phosphatases with the CX_5_RT/S motif in their active site and with activity toward more than pTyr substrates are categorized as ***dual-specific PTPs*** (DUSPs). DUSPs are the most diverse subclass among cysteine-based PTPs in terms of their substrate specificity. As described in more detail in the reviews of Alonso et al. (2004) and Tautz et al. (2013), they comprise phosphatases with activity toward protein- and/or non-protein substrates (**Figure 1**). Examples for protein-specific DUSPs are the phosphothreonine (pThr)-/pTyr-specific MAPK phosphatases (MKPs), the pSer-specific slingshots (SSHs), and the pSer-/pThr-specific Cdc14s.

Several DUSPs dephosphorylate more than protein-derived substrates. Examples for these phosphatases are the mRNA-specific phosphatase DUSP11 (also referred to as PIR1; Yuan et al., 1998), the phosphoinositol-specific PTENs (phosphatase and tensin homologs), MTMs (myotubularins) and INPP4s (inositol-4-phosphatases; Liu and Bankaitis, 2010; Kim et al., 2013), and the carbohydrate-specific phosphatase laforin (Ganesh et al., 2000).

For the following discussion, we selected only a few examples to illustrate general features of DUSPs regarding their structural, catalytic, and functional properties. We are aware that this can just give a rough insight into this field, because of the huge amount of information being available about this subclass of enzymes.

The first member of DUSPs described was VH1 from the vaccinia virus (Guan et al., 1991). This enzyme dephosphorylates pSer, pThr, and pTyr residues *in vitro* and *in vivo* (Guan and Dixon, 1991; Derrien et al., 1999). Included in the encapsidated fluid of vaccinia virions, VH1 is released into the intracellular space during the infection of a cell. Here, it dephosphorylates Stat1, an effector molecule of the host cell, which otherwise would initiate the antiviral immune response (Najarro and Traktman, 2001; Mann et al., 2008). In addition to that, VH1 activates own viral proteins by dephosphorylating them during the infection of a cell, i. a. those ones that mediate the viral morphogenesis (Liu et al., 1995; Derrien et al., 1999). In this way, VH1 is crucial for maintaining the viability of the virus.

The first homolog to VH1 found in humans was VHR (Ishibashi et al., 1992). This phosphatase hydrolyzes pTyr-/pThr-residues derived from MAP-kinases, such as Erk and Jnk and, thus, downregulates signaling cascades that are associated with cell proliferation and differentiation (Ishibashi et al., 1992; Denu et al., 1995b; Todd et al., 1999; Alonso et al., 2001).

Another human VH1-related phosphatase, DUSP11, specifically dephosphorylates mRNA and shows less activity toward phosphoproteins. Because DUSP11 interacts with the RNA/ribonucleoprotein-complex, it is proposed to be involved in the cellular mRNA metabolism (Yuan et al., 1998; Deshpande et al., 1999; Sankhala et al., 2014).

One of the most prominent members of non-protein-specific DUSPs is the tumor suppressor phosphatase PTEN. This enzyme was initially identified by screening diverse tumor-derived tissues as one of the most frequently mutated proteins (Li et al., 1997). Based on the P-loop motif in its active site, the cytosolic phosphatase was primarily believed to be a cysteine-based PTP specific for phosphorylated protein substrates. Shortly after its discovery, it has been demonstrated that PTEN is indeed a dual-specific class I PTP with dephosphorylation activity toward pTyr, pSer, and pThr residues *in vitro* (Furnari et al., 1997; Li and Sun, 1997; Myers et al., 1997). However, follow-up studies demonstrated that PTEN’s main substrate preference *in vivo* is toward the 3′-position of the phosphoinositide (PI) species PI(3,4,5)P_3_ at the inner cell membrane surface (Myers et al., 1997, 1998). Thus, PTEN counteracts the PI3K-signaling cascade, which otherwise leads to unrestricted cell growth with significantly increased susceptibility for tumor genesis (Knobbe et al., 2002; Leslie and Downes, 2002; Zhang and Yu, 2010; Miller et al., 2011).

One last example for DUSPs should be mentioned here: laforin. This human enzyme is the only phosphatase known so far that dephosphorylates complex carbohydrates (Worby et al., 2006; Tagliabracci et al., 2007). Laforin is further capable to dephosphorylate pTyr, pSer, and pThr residues *in vitro* (Ganesh et al., 2000) and pSer *in vivo* (Lohi et al., 2005). Missense mutations in the respective gene, *EMP2A*, which disrupt the activity of the phosphatase, are associated with a fatal progressive autosomal recessive form of myoclonic epilepsy, the Lafora disease (Tagliabracci et al., 2007). The mechanism by which laforin prevents the disease is only poorly understood. First results indicate that the phosphatase intervenes in the glycogen metabolism and prevents the formation of polyglucosan inclusion bodies in the cytosol of almost all cell types. These so-called “Lafora bodies” are a typical symptom of the disease (Wang et al., 2002; Fernández-Sánchez et al., 2003; Tagliabracci et al., 2007; Roach et al., 2012).

All these facts illustrate the broad substrate spectrum of DUSPs and their crucial relevance in human physiology. For further information about these phosphatases, the reader is referred to the detailed reviews listed here (Tronchère et al., 2003; Pulido and van Huijsduijnen, 2008; Liu and Bankaitis, 2010; Pulido et al., 2013; Tautz et al., 2013; He et al., 2014).

It should be noted, that the similarity of classical, dual-specific, and non-protein-/dual-specific phosphatases is largely restricted to the P-loop in the active site and the conservation of the folding pattern of the catalytic domain. The domains and regions that flank the phosphatase domain are diverse among class I PTPs, and specify their differences in cellular localization, activation, and regulation.

#### Class II PTPs – the low molecular weight PTPs

Class II PTPs are the so-called “low molecular weight” PTPs (LMW-PTPs). With ∼15-18 kDa, these phosphatases are smaller than class I PTPs that have a molecular weight of at least ∼30–50 kDa. Due to their catalytic activity at pH values ≤6, LMW-PTPs have also been classified as acid phosphatases (ACPs; Hopkinson et al., 1963; Lawrence and Van Etten, 1981; Lucentini et al., 2003). LMW-PTPs are soluble phosphatases with substrate specificity for pTyr substrates (Zhang and Van Etten, 1991; Stefani et al., 1993), but not for pSer or pThr residues (Ramponi et al., 1989; Wo et al., 1992).

Originally isolated from human red cells (Hopkinson et al., 1963), LMW-PTPs have been found in numerous prokaryotes and eukaryotes (Ramponi and Stefani, 1997a; Raugei et al., 2002). In mammals, they are ubiquitously expressed with no particular tissue-specific distribution (de Araujo et al., 1976; Zhang and Van Etten, 1990; Ramponi and Stefani, 1997b). Up to now, four human isoforms have been identified. All of them are splice variants of the transcription product of the *ACP1* gene (Raugei et al., 2002). Two catalytically active isoforms, IF1 and IF2 (also named as HCPTPA and HCPTPB), differ only in a short sequence that spans one of the loop regions surrounding the active site. This variation in structure causes differences in both, substrate specificity and binding affinities against modulating ligands (Wo et al., 1992; Cirri et al., 1996; Ramponi and Stefani, 1997a). Apart from that, two other human splice variants of the *ACP1* transcription product have been described, SV3 and LMW-PTP-C. Both isoforms seem to be catalytically inactive, but might regulate the active isoenzymes through the competition for substrate binding (Modesti et al., 1998; Tailor et al., 1999).

In mammals, catalytically active LMW-PTPs downregulate the activity of several tyrosine kinase receptors, for example the insulin receptor (Chiarugi et al., 1997), the vascular endothelial (Huang et al., 1999), the epidermal (Ramponi et al., 1989), and the fibroblast growth factor receptor (Park et al., 2002). Moreover, LMW-PTPs also regulate the cellular reaction to platelet-derived growth factor stimulation (Chiarugi et al., 2000). Because LMW-PTPs are involved in a variety of signaling cascades, the reduction or loss of their activity leads to different pathologic manifestations (Bottini et al., 2002), including allergy and asthma (Bottini et al., 2007), autoimmune diseases (Bottini et al., 2000), and neurodegenerative disorders like the Alzheimer’s disease (Shimohama et al., 1993, 1995). In particular, since LMW-PTPs play a crucial role in mitogenic signaling pathways, their malfunction entails high oncogenic potential (Malentacchi et al., 2005; Alho et al., 2008). Therefore, LMW-PTPs form another class of target molecules for therapeutics against serious diseases, including cancer (Homan et al., 2010; Mascarello et al., 2010; Seiler et al., 2013).

#### Class III PTPs – the Cdc25 phosphatases

Class III PTPs belong to the family of cell division control proteins (Cdc). According to this nomenclature, class III PTPs are named as Cdc25. Initially characterized in yeast (Russell and Nurse, 1984), these phosphatases are expressed in all eukaryotes (Boutros et al., 2007; Rudolph, 2007) except plants (Boudolf et al., 2006). Up to now, three isoforms have been identified in humans: Cdc25A, B and C (Galaktionov and Beach, 1991).

Cdc25 isoforms specifically dephosphorylate cyclin-dependent kinases (Cdks) at their inhibitory, dually phosphorylated N-terminal Thr-Tyr motif (Gautier et al., 1991; Krek and Nigg, 1991; Honda et al., 1993). In turn, these phosphatases show dual-specific substrate specificity. Cdks require for their activation the dephosphorylation by Cdc25 and, additionally, a phosphorylation mediated by Cdk-activating kinases (Kaldis, 2014). Through this enzymatic interplay, Cdks initiate the progression through the cell cycle (Gautier et al., 1991; Honda et al., 1993; Lammer et al., 1998). Apart from that, Cdc25s are also involved in preventing Cdk activation upon cellular DNA damage or incomplete replication (Kristjánsdóttir and Rudolph, 2004; Karlsson-Rosenthal and Millar, 2006). Acting as regulator of the eukaryotic cell cycle qualifies Cdc25 phosphatases as promising targets for anti-cancer therapeutics (Kristjánsdóttir and Rudolph, 2004; Rudolph, 2007).

Because Cdc25s dephosphorylate more than pTyr substrates, they are often called dual-specific phosphatases. This nomenclature might cause confusion by mixing up Cdc25s with dual-specific phosphatases from class I PTPs. Here, it should be noted, that despite the activity toward more than pTyr substrates, dual-specific PTPs of class I and III are individual subfamilies with significant differences in protein structure.

Interestingly, Cdc25s have evolved as a unique subclass of PTPs, but the reason for this uniqueness has been controversially discussed. As mentioned by Alonso et al. (2004), one possible explanation could be that ancestral Cdc25s had already acted as rhodanese-type enzymes – a class of sulfur-transfer proteins (Cipollone et al., 2008) – on Cdks, but were finally transformed into cysteine-based PTPs when the Tyr/Thr phosphorylation of Cdks appeared. This idea is supported by the fact that the overall structure of Cdc25s is rather similar to rhodanese enzymes than to class I and II PTPs (Fauman et al., 1998; Reynolds et al., 1999; Bordo and Bork, 2002).

Although all three subclasses I to III of the cysteine-based PTP family share similarities regarding their catalytic mechanism and active site structure, all of them have evolved independently from each other (Alonso et al., 2004; Moorhead et al., 2009). From that, the question arises: to which subclass of PTPs does the family of VSPs belong?

### INTEGRATION OF VOLTAGE SENSITIVE PHOSPHATASES INTO THE CLASSIFICATION SYSTEM OF PTPs

The first mammalian variants of VSPs were described in the early 2000s with publications about transmembrane phosphatases with tensin homology (TPTEs^2^; Chen et al., 1999; Guipponi et al., 2000, 2001; Walker et al., 2001; Wu et al., 2001; Tapparel et al., 2003). In these early studies, the research had been focused only on the biochemical characterization of the enzymatic activity, because the proteins share a high sequence homology with the tumor suppressor PTEN. Finally, bioinformatic analyses suggested that the transmembrane part of these phosphatases might act as voltage sensor domain (Kumanovics et al., 2002). However, no functional characterization of a voltage-dependent activity was carried out at this point. With the description of Ci-VSP (VSP from *Ciona intestinalis*) in Murata et al. (2005), the first experimental evidence for the existence of a transmembrane phosphatase with voltage-driven activity was published.

Ci-VSP, the most prominent example of VSPs to date, can be easily expressed in heterologous expression systems; a fact that facilitates the electrophysiological characterization of this protein. As other VSPs, Ci-VSP consists of a membrane-spanning voltage sensor domain and a cytosolic catalytic domain. The voltage sensor domain consists of four putatively α-helical transmembrane segments (Li et al., 2014). The fourth segment contains positively charged residues arranged in a periodical order as it can be found in voltage-gated ion channels (Villalba-Galea, 2012b). The cytosolic domain consists of two subdomains, the phosphatase and an adjacent C2 domain (Matsuda et al., 2011; Liu et al., 2012). The phosphatase-/C2-complex of Ci-VSP shows ∼40% amino acid homology to the tumor suppressor PTEN. Due to this fact, the phosphatase domain of Ci-VSP is structurally related to that one of class I PTPs (Okamura and Dixon, 2011; Villalba-Galea, 2012a).

Generally, VSPs are capable to “translate” voltage stimuli at the plasma membrane directly into enzymatic activity in the cytosol. Positive membrane potentials lead to conformational changes in the voltage sensor domain that activate the intracellular catalytic domain. In the activated state, the enzymatic domain cleaves phosphate groups from the inositol ring of phosphoinositide phosphates (PIPs). In particular, Ci-VSP specifically hydrolyzes the 5′-phosphate of the PIP-species PI(3,4,5)P_3_ and PI(4,5)P_2_ (Iwasaki et al., 2008; Halaszovich et al., 2009). Furthermore, several results indicated a 3′-site activity toward PI(3,4)P_2_ for Ci-VSP when the phosphatase was expressed in *Xenopus* oocytes, but with a decrease in efficiency compared to the 5′-activitiy (Sakata et al., 2011; Sakata and Okamura, 2013). Controversially, such a 3′-specificity could not be detected when Ci-VSP was expressed in mammalian cells (Lacroix et al., 2011; Halaszovich et al., 2012). This discrepancy raised the question whether the 3′-site activity of Ci-VSP is specific for the oocyte expression system (Halaszovich et al., 2012).

After the discovery of Ci-VSP, several homologs have been described from other species, such as zebrafish (*Danio rerio*, Dr-VSP; Hossain et al., 2008), chicken (*Gallus gallus*, Gg-VSP; Neuhaus and Hollemann, 2009; Kurokawa et al., 2012), the African clawed frog (*Xenopus laevis*, Xl-VSP; Ratzan et al., 2011), and urodele amphibians (Mutua et al., 2014). In addition, transmembrane phosphatases found in mammals have been characterized in more detail in the recent years (Halaszovich et al., 2012; Kurokawa et al., 2012; Sutton et al., 2012). Although several studies demonstrated the expression of VSPs in testis, intestine, stomach, kidney, and the nervous system (Chen et al., 1999; Walker et al., 2001; Tapparel et al., 2003; Murata et al., 2005; Neuhaus and Hollemann, 2009; Ogasawara et al., 2011), the physiological role of VSPs in their native organisms is still elusive.

There are several isoforms of VSPs that are catalytically inactive toward their presumed PIP-substrates, for example TPTE/hVSP2 (Walker et al., 2001; Leslie et al., 2007). However, all active isoenzymes share the common property of dephosphorylation activity at positive membrane potentials. Moreover, all homologs characterized so far show substrate specificity against PIP-lipids, whereas the preference either for the 3′- or the 5′-phosphate at the inositol ring seems to differ among VSPs (Walker et al., 2001; Murata et al., 2005; Iwasaki et al., 2008; Halaszovich et al., 2009, 2012; Lacroix et al., 2011; Kurokawa et al., 2012).

In conclusion, because of the CX_5_RS/T motif in the active site, VSPs belong to the family of cysteine-based PTPs. Furthermore, the catalytic domain of VSPs shows a three-dimensional folding that is characteristic for class I PTPs. Together with the fact that VSPs specifically dephosphorylate PIP-lipids, the criteria are met to classify VSPs as dual-specific class I PTPs (**Figure 1**).

## GENERAL FOLDING OF THE CATALYTIC DOMAIN OF PTPs

**FIGURE 2:**
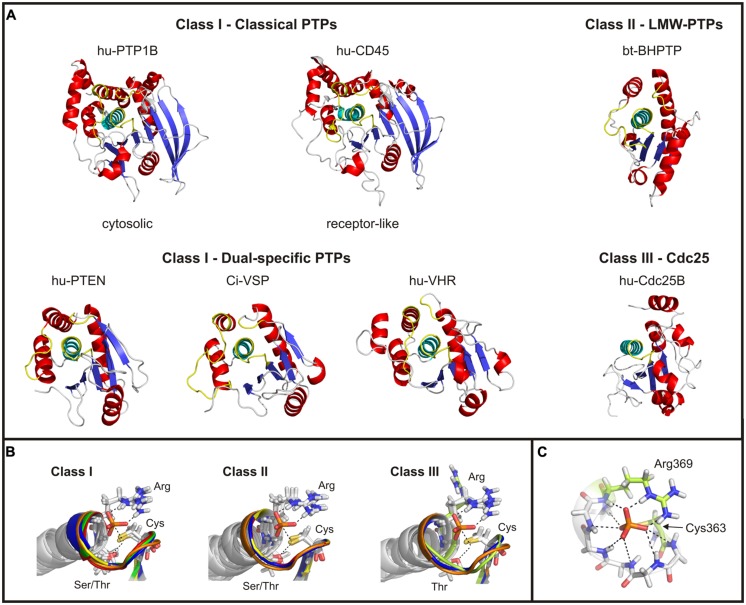
**Structural comparison of the catalytic domains of cysteine-based PTPs. (A)** Catalytic domains of exemplary chosen phosphatases are shown. The twisted β-sheet core is colored in blue; the α-helices surrounding the core are shown in red. The α-helix C-terminal to the active site loop is depicted in cyan, and the regions corresponding to the P-, TI- and WPD-loop of class I PTPs in yellow (see text for details). In case of Cdc25s, no TI- and WPD-loop exist. The structure of Ci-VSP was taken from the MD simulation (Hobiger et al., 2013) which was performed on the basis of the crystal structure by Matsuda et al. (2011; PDB accession number 3awe). All other structures were created based on the crystal structures available in the RCSB protein data bank. The accession numbers are: 1nwe (hu-PTP1B), 1ygu (hu-CD45. Here, the catalytically active domain D1 is shown.), 1d5r (hu-PTEN), 1vhr (hu-VHR), 1dg9 (bt-BHPTP), and 1qb0 (hu-Cdc25B). Abbreviations are: hu, human; bt, *bos taurus*; and BHPTP, bovine heart PTP. **(B)** The P-loop motif and the following α-helix are structurally aligned for all classes of cysteine-based PTPs. For comparable reasons, the P-loop structure of Ci-VSP has been integrated in all panels (colored in orange). Class I PTPs used for the alignment were the human isoforms of PTP1B (1nwe: green), of the D1 domain of CD45 (1ygu, red), of PTEN (1d5r, blue), and of VHR (1vhr, yellow). Class II PTPs are the bovine BHPTP (1dg9, yellow), and the human isoforms HCPTPA (5pnt, blue) and HCPTPB (1xww, red). Class III PTPs are the human isoforms Cdc25A (1c25, yellowish) and Cdc25B (1qb0, blue). In all structures a phosphate anion is indicated in the active site. **(C)** The phosphate group of the substrate is coordinated by hydrogen bonds with backbone atoms of the P-loop residues, as demonstrated here for Ci-VSP. Residues crucial in catalysis, Cys363 and Arg369, are particularly denoted.

**FIGURE 3:**
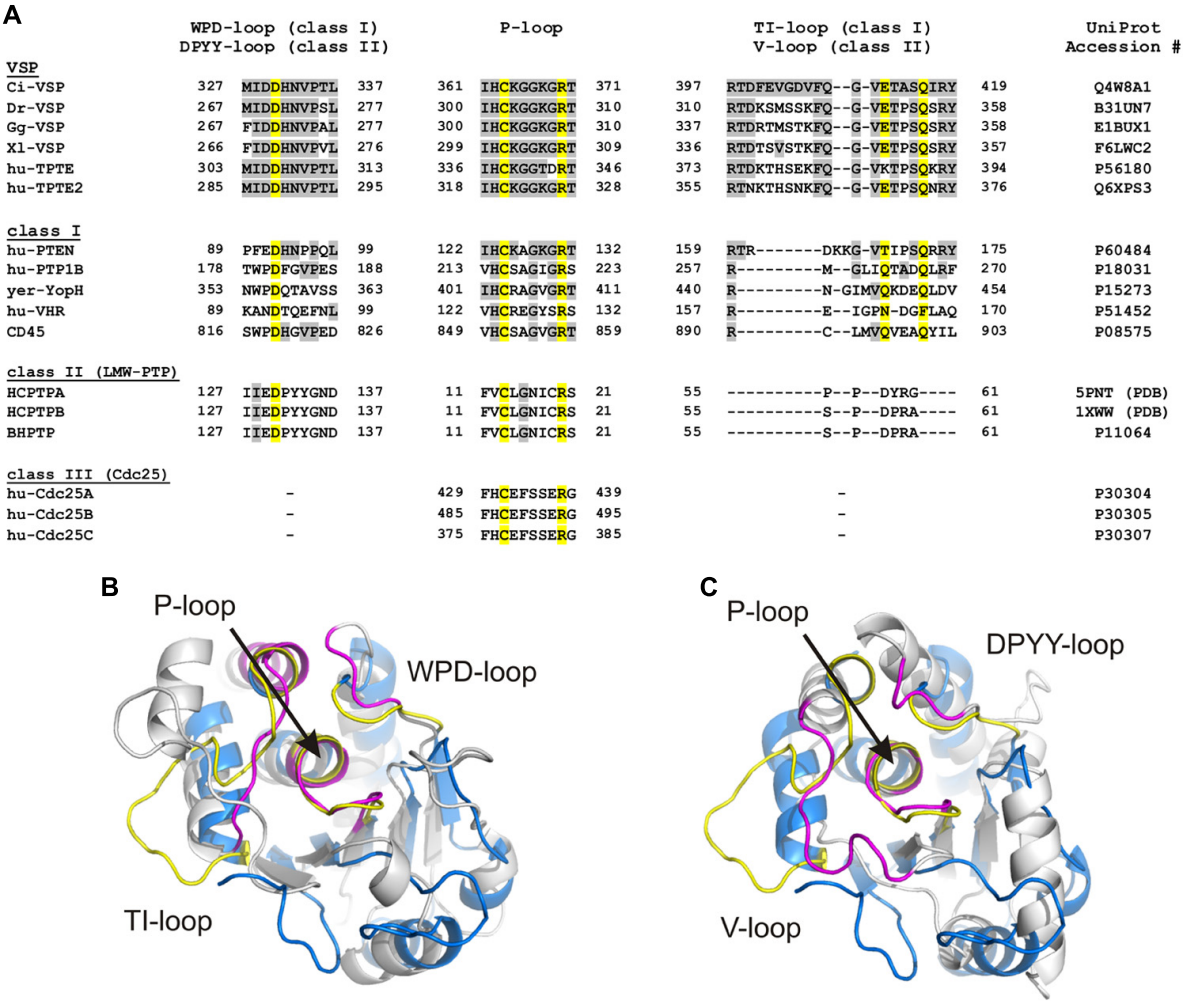
**Loop regions determine the conformation of the substrate binding pocket in cysteine-based PTPs. (A)** Sequence alignment of the three loops which specify the conformation of the substrate binding pocket. The P-loop contains the highly conserved CX_5_R motif that serves as active site. The WPD-/DPYY- and the TI-/V-loops form the side walls of the substrate binding pocket in class I and II PTPs. Because class III PTPs lack the two latter loops, no sequence alignment could be performed for these regions. Amino acids identified as identical to Ci-VSP are marked in gray. Yellow colored residues are crucial for catalysis and widely conserved among PTPs. UniProt accession numbers are indicated for all sequences used for the alignment; in case of HCPTPA and HCPTPB, accession numbers in the PDB database are given, since UniProt contains the same reference sequence for both PTPs. **(B,C)** Structural alignment of the phosphatase domain of Ci-VSP (colored in blue) with **(B)** the catalytic domain of the human class I PTP, VHR (PDB accession number 1vhr) and **(C)** the class II PTP, BHPTP (bovine heart PTP, PDB 1dg9). VHR and BHPTP are colored in gray. Loops are highlighted in yellow for Ci-VSP and in magenta for the respective PTP. The catalytic domain of Ci-VSP was obtained by MD simulations (Hobiger et al., 2013) based on the crystal structure of Matsuda et al. (2011; PDB 3awe).

The catalytic domains of cysteine-based PTPs have various folding properties in common. For example, all of them contain a central core consisting of highly twisted β-sheets, which are flanked by several α-helices (**Figure 2A**). One of the β-sheet-α-helix-loops contains the active site motif CX_5_RS/T. This P-loop is highly conserved and shares a homolog folding pattern for all cysteine-based PTPs (**Figure 2B**). Although the residues in the X_5_-segment vary largely between the different PTP subclasses (**Figure 3A**), the superposition of the P-loop structures evinces only minor deviations in the C_α_-trace of the protein backbones of less than 1 Å.

For all cysteine-based PTPs, the active site is located within a crevice with the conserved cysteine at its bottom. Together with the P-loop arginine, the catalytically active residue forms a cradle-like structure (**Figure 2B**). This conformation is important for the correct binding of the substrate and for stabilizing the active site structure during the whole catalytic cycle. A network of hydrogen bridges between the phosphate group of the substrate and the backbone atoms of the P-loop further facilitates this stabilization (**Figure 2C**).

The active site pocket of class I and II PTPs is flanked by two loop regions, which are named TI- and WPD-loop for class I PTPs, and variable (or V-) and DPYY-loop for class II PTPs (**Figure 3**). In both classes, these regions contain amino acids that are crucial for the enzymatic reaction. Although the loop regions in class I and II PTPs differ in length and sequence (**Figure 3A**), their existence indicates a common principle for cysteine-based PTPs regarding the regulation of their activity.

In class III PTPs, none of these two loops exists (**Figure 2A**). However, these phosphatases use mechanisms that compensate for the lack of the loop structures flanking the active site. This will be discussed later in this review.

Despite several similarities, class I to III PTPs show significant structural differences which will be discussed in the following.

### THE CATALYTIC DOMAIN OF CLASS I PTPs

Catalytic domains of classical PTPs contain ∼240–280 amino acids, and are the largest among the cysteine-based PTPs (**Figure 2A**). The catalytic domains of dual-specific PTPs are smaller; for example, the phosphatase of PTEN contains ∼180 amino acids. In particular, class I PTPs differ in the amount of β-sheets in the protein core and of the α-helices flanking the core. These structural variations are one of the reasons for differences in regulation of the catalytic activity and the cellular targeting of class I PTPs (Tabernero et al., 2008).

Each class I PTP contains at least one catalytic domain. Examples for single phosphatase PTPs are PTP1B, VHR, laforin and PTEN (Tonks, 2006; Pulido et al., 2013). Most of the receptor-like PTPs contain two tandemly repeated catalytic domains, called D1 and D2 (Alonso et al., 2004), where the latter is located more distally to the membrane and has no catalytic activity (Streuli et al., 1990). As shown for CD45, the D2 domain is proposed to play a regulatory role for the phosphatase activity of D1 (Nam et al., 2005).

Another characteristic feature that differs among class I PTPs is the depth of the substrate binding pocket, with ∼9 Å for classical and ∼6 Å for dual-specific PTPs (Barford et al., 1994; Stuckey et al., 1994; Yuvaniyama et al., 1996). This difference is proposed to determine the substrate specificity of the phosphatases (Barford et al., 1994; Jia et al., 1995; Yuvaniyama et al., 1996). The deeper pocket in classical PTPs permits the access to the active site only for longer pTyr residues, whereas dual-specific phosphatases are capable to bind pSer, pThr, and/or pTyr residues.

In case of the PIP-specific phosphatase PTEN, a crevice of 8 Å in depth was observed in the crystal structure with a wider access than in classical PTPs. This structural feature was suggested to be the reason that PTEN preferentially accommodates the sterically demanding PIP-substrates in its active site instead of pTyr, pSer, or pThr substrates (Lee et al., 1999).

### THE CATALYTIC DOMAIN OF CLASS II PTPs

LMW-PTPs contain one catalytic domain that consists of four β-sheets flanked by at least five α-helices on both sides (**Figure 2A**; Su et al., 1994; Zhang et al., 1994a). In contrast to class I PTPs, the P-loop is located at the N-terminal part of the phosphatase connecting sheet β_1_ with helix α_1_. For example, the catalytically active cysteine of the bovine liver LMW-PTP is located at position 12 in contrast to the soluble PTP1B, a class I PTP, which has the cysteine at position 215 (**Figure 3A**). As mentioned above, the P-loop in LMW-PTPs is surrounded by two loops: (i) the V-loop, which differs in length and sequence among the phosphatases, and (ii) the DPYY-loop. Both loops are as closely positioned to the active site as the TI- and WPD-loops in class I PTPs (**Figures 3B,C**). This configuration leads to a compact substrate binding pocket in LMW-PTPs, which is smaller, but similar to that of class I PTPs. This is an interesting fact, since the overall amino acid sequences of both PTP subclasses show no significant homology except for the P-loop motif (**Figure 3A**; Su et al., 1994).

### THE CATALYTIC DOMAIN OF CLASS III PTPs

The full length of Cdc25 phosphatases ranges from 300 to 600 amino acids (Kristjánsdóttir and Rudolph, 2004). Structurally, the enzymes are divided into an N- and C-terminal region. The N-terminal part is highly variant among Cdc25 phosphatases and contains several regulatory sites, which are involved in interactions with other proteins (Conklin et al., 1995; Chen et al., 2000a; Gnesutta et al., 2001; Kawabe et al., 2002), in phosphorylation or ubiquitination (Hoffmann et al., 1993, 1994; Gabrielli et al., 1997; Bernardi et al., 2000; Mailand et al., 2000; Donzelli et al., 2002).

The C-terminal catalytic domain consists of about 200 residues. It contains the active site motif CX_5_R, but lacks the serine or threonine seven positions more C-terminal to the catalytically active cysteine as found in other PTPs (**Figure 3A**). However, the motif adopts the cradle-shaped conformation that is typical for cysteine-based PTPs, with the cysteine and the arginine at positions that allow for binding of the substrate in the active site and for passing through the catalytic cycle (**Figure 2B**; Fauman et al., 1998; Reynolds et al., 1999). Here, it should be noted that in the crystal structure of Cdc25A (Fauman et al., 1998), the arginine points away from the substrate binding pocket (**Figure 2B**); this configuration would be unfavorable for substrate binding. However, in a structure subsequently described for the Cdc25A-homolog Cdc25B, the arginine was positioned in a way that resembles that of other PTPs (Reynolds et al., 1999). Additionally, molecular dynamics (MDs) simulations carried out on Cdc25A in presence of an oxyanion revealed a spontaneous flip of the P-loop segment during docking of the ligand into the substrate binding pocket. In this way, the active site of Cdc25A adopts a conformation similar to other PTPs (Kolmodin and Åqvist, 2000).

Apart from the active site motif, the α-helix following C-terminally after the P-loop in Cdc25 matches well with the configuration found in class I and II PTPs (**Figure 2B**). However, the catalytic domain of Cdc25 contains a smaller number of β-strands and α-helices compared to class I PTPs (**Figure 2A**). Moreover, the loop regions surrounding the substrate binding pocket in class I and II PTPs are absent in Cdc25 phosphatases. Interestingly, the overall structure of class III PTPs is less homologous to other cysteine-based PTP subfamilies, but shows more similarities to their ancestral relatives, the rhodanese enzymes (Fauman et al., 1998; Reynolds et al., 1999).

## GENERAL CATALYTIC MECHANISM OF PTPs

**FIGURE 4:**
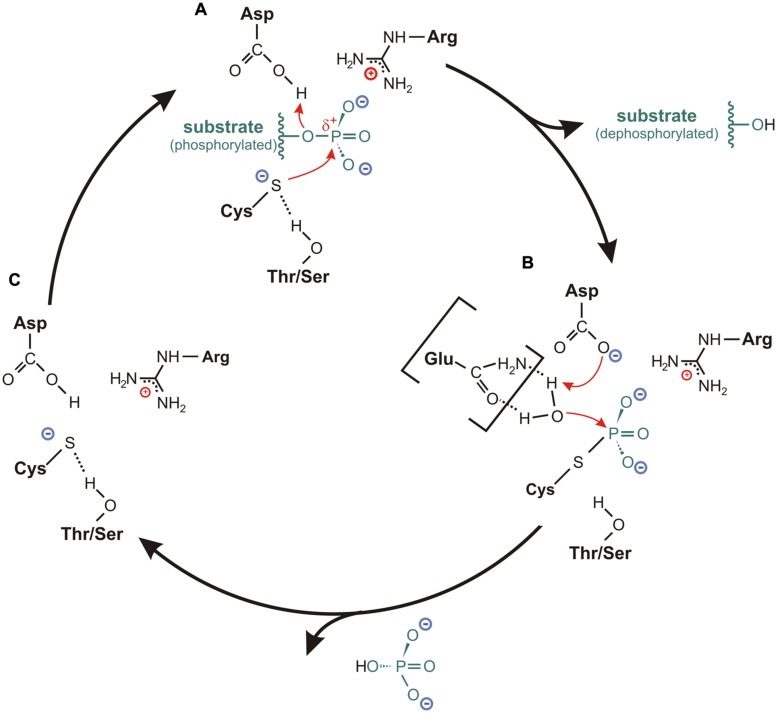
**Catalytic cycle for cysteine-based PTPs. (A)** In the resting state, the sulfur of the catalytic cysteine is stabilized in an anionic thiolate form. This is mediated by a hydrogen bond between the sulfur and the hydroxyl oxygen of the conserved Thr-/Ser-side chain located in the P-loop. Upon binding of the substrate, where the P-loop arginine is particularly involved in the positioning of the substrate, the thiolate acts as catalytic nucleophile by attacking the phosphorus atom of the substrate. The formation of a cysteinyl-phosphate intermediate is facilitated by a general acid (usually an aspartate from the WPD- or DPYY-loop flanking the active site), which donates a proton to the substrate leaving group. Subsequently, the dephosphorylated substrate dissociates from the substrate binding pocket. **(B)** The hydrolysis of the cysteinyl-phosphate intermediate is mediated by an activated water molecule. Here, the aspartate or a corresponding residue serves as general base by accepting one proton of the water molecule. In several class I PTPs, the water molecule is activated through hydrogen bonds with one or two polar amino acids in the TI-loop, as it is indicated here for an interaction with a glutamine. After accepting the proton from the general base, the phosphate dissociates from the catalytic cysteine, so that **(C)** the resting state conformation of the active site is reconfigured.

Since the three-dimensional structure of the active site is similar for all three PTP subclasses, it is reasonable to assume that these phosphatases share a similar enzymatic reaction mechanism. Indeed, results from numerous studies about different cysteine-based PTPs converge on the same general two-step catalytic mechanism that applies for all class I to III phosphatases (**Figure 4**; Zhang et al., 1994c; Denu and Dixon, 1995, 1998; Tonks and Neel, 2001; Rudolph, 2002; Wang et al., 2003).

The reaction cycle starts with a nucleophilic attack on the phosphorus atom of the substrate; a step that is mediated by the cysteine in the PTP signature motif (**Figure 4A**). Here, a general acid located in close vicinity to the catalytic cysteine donates a proton to the substrate leaving group. In this way, a cysteinyl-phosphate intermediate is formed and the dephosphorylated substrate dissociates from the active site cavity (**Figure 4A**; Guan and Dixon, 1991; Jia et al., 1995; Denu et al., 1996; Pannifer et al., 1998).

The second reaction step requires the presence of an activated water molecule. This is achieved by a general base (**Figure 4B**). The residue that serves as general acid during the first reaction step usually acts as general base in this second step by accepting a proton from the activated water molecule (Taddei et al., 1994; Denu et al., 1995a, 1996; Flint et al., 1997; Pannifer et al., 1998). The resulting configuration in the active site promotes the hydrolysis of the cysteinyl-phosphate intermediate, whereby the phosphate group is released. This step finalizes the reaction and restores the conformation of the active site for a new catalytic cycle (**Figure 4C**).

### DIFFERENCES IN THE CATALYTIC CYCLE BETWEEN CLASS I, II, AND III PTPs

A close look into the reaction scheme of cysteine-based PTPs implies that not only the active site motif participates in catalysis, but that all conserved loop regions are involved in the reaction. In particular, for class I and II PTPs, the WPD- or DPYY-loop contains the general acid for the first reaction step (**Figure 3A**). Due to the lack of this loop in class III PTPs, the existence of a general acid has been controversial. An early study indicated that an aspartate localized in the protein apart from the P-loop serves as general acid in Cdc25 (Eckstein et al., 1996). However, the crystal structure of the human isoform Cdc25A had been resolved shortly afterwards, and it demonstrated a structural rather than a catalytic role for this aspartate (Fauman et al., 1998). Based on this and the Cdc25B structure, one of the two glutamates found in the X_5_-segment of the active site motif (**Figure 3A**) were proposed to act as general acid (Fauman et al., 1998; Reynolds et al., 1999). These results were further supported by MD simulations carried out on Cdc25B in presence of its natural substrate (Arantes, 2008). In contrast to that, it has been further suggested that Cdc25 phosphatases might not require a general acid, because changes in pH in the active site during the catalytic cycle could compensate for the lack of a proton donor (Rudolph, 2002, 2007). Controversially, several studies imply that the general acid might be provided by the substrate itself instead of being contributed by the phosphatase (Chen et al., 2000b). However, up to now this question has not been completely resolved.

For the second catalytic step in class I and II PTPs, the amino acid that served as general acid in the previous reaction step is now involved in the activation of a water molecule by acting as general base. A different mechanism can be assumed for class III PTPs again, because of the lack of the WPD- or a WPD-equivalent loop. Studies addressing the influence of the pH on the active site suggest that, also here, the two glutamates in the P-loop might be involved in the activation of a critical water molecule (Rudolph, 2002, 2007). However, also this issue is still under controversial discussion.

In the following, we want to elucidate in more detail the role of the loop regions surrounding the active site. Because Cdc25 phosphatases are such a distinct subclass of cysteine-based PTPs with large differences in the amino acids that might participate in the catalytic reaction, we will exclude them from further discussion. Readers interested in details of the catalytic mechanism of class III PTPs are referred to the reviews of Rudolph (2002, 2007).

## STRUCTURAL ELEMENTS DEFINING THE PHOSPHATASE ACTIVITY OF PTPs

### THE P-LOOP

As mentioned above, the active site motif CX_5_RS/T is highly conserved among all cysteine-based PTPs (**Figure 3A**). This loop region is located at the bottom of the substrate binding pocket and is surrounded by other loop regions forming the side walls of the pocket. Polar or charged residues in the P-loop are involved in substrate docking and in the stabilization of the cysteinyl-phosphate intermediate during the catalytic cycle. In the following, we will discuss the functional relevance of critical amino acids in this loop region.

#### The catalytic cysteine

The cysteine within the CX_5_RS/T-motif of the P-loop is the most crucial residue for the catalytic reaction. The exchange of this residue by serine results in the inactivation of all cysteine-based PTPs. The cysteine-to-serine substitution in the active site is one of the best characterized mutations of PTPs, since it is generally used as negative control to analyze the catalytic activity of these enzymes.

Apart from that, it has to be considered that the nucleophilic attack mediated by the P-loop cysteine requires a correct positioning of the sulfhydryl-side chain with respect to the size of the substrate binding pocket and the adjacent residues being involved in the reaction steps. In this context, it has been frequently described that the nucleophilic attack requires the sulfur of the cysteine to be in an anionic thiolate configuration (Zhang, 1993; Denu and Dixon, 1995; Evans et al., 1996; Lohse et al., 1997). Crystal structures or results obtained by MD simulations showed that this configuration is enabled by a hydrogen bond between the sulfur of the cysteine and the hydroxyl oxygen of the conserved P-loop threonine or serine seven positions C-terminally to the cysteine (**Figures 2B**,**3A**,**4A**; Barford et al., 1994; Stuckey et al., 1994; Yuvaniyama et al., 1996; Barford et al., 1998). As proposed by Evans et al. (1996) for BHPTP (a class II PTP), the hydrogen bond between the cysteine and the serine causes an unusually low pK_a_ of the sulfhydryl-side chain, with less than 4. Similarly, reduced pK_a_ values were also described for other cysteine-based phosphatases, such as PTP1B (Lohse et al., 1997) or YopH from *Yersinia pestis* (Zhang, 1993). This condition is proposed to stabilize the cysteine in the thiolate configuration, so that the nucleophilic attack on the substrate can proceed (Dillet et al., 2000). In addition to that, Denu and Dixon (1995) proposed that the hydrogen bond between the cysteine and the Thr/Ser might not only stabilize the conformation of the enzyme’s resting state, but also that one of the cysteinyl-phosphate intermediate. In this way, the hydrolysis of the phosphate during the second reaction step might be facilitated (Denu and Dixon, 1995).

Analogously in Ci-VSP, the catalytic cysteine and the P-loop threonine are closely located to each other (**Figure 2A**); a configuration that was found in crystal structures of the cytosolic domain of Ci-VSP (Matsuda et al., 2011; Liu et al., 2012) and in a structural model of the protein obtained by MD simulations (Hobiger et al., 2013). These results suggest the existence of a hydrogen bond between the two residues also for Ci-VSP. However, it is still elusive if this configuration has any influence on the pK_a_ value of the cysteine side chain. Therefore, the question remains if the sulfur is in an anionic thiolate form in VSPs, and which role this configuration plays in catalysis.

**FIGURE 5:**
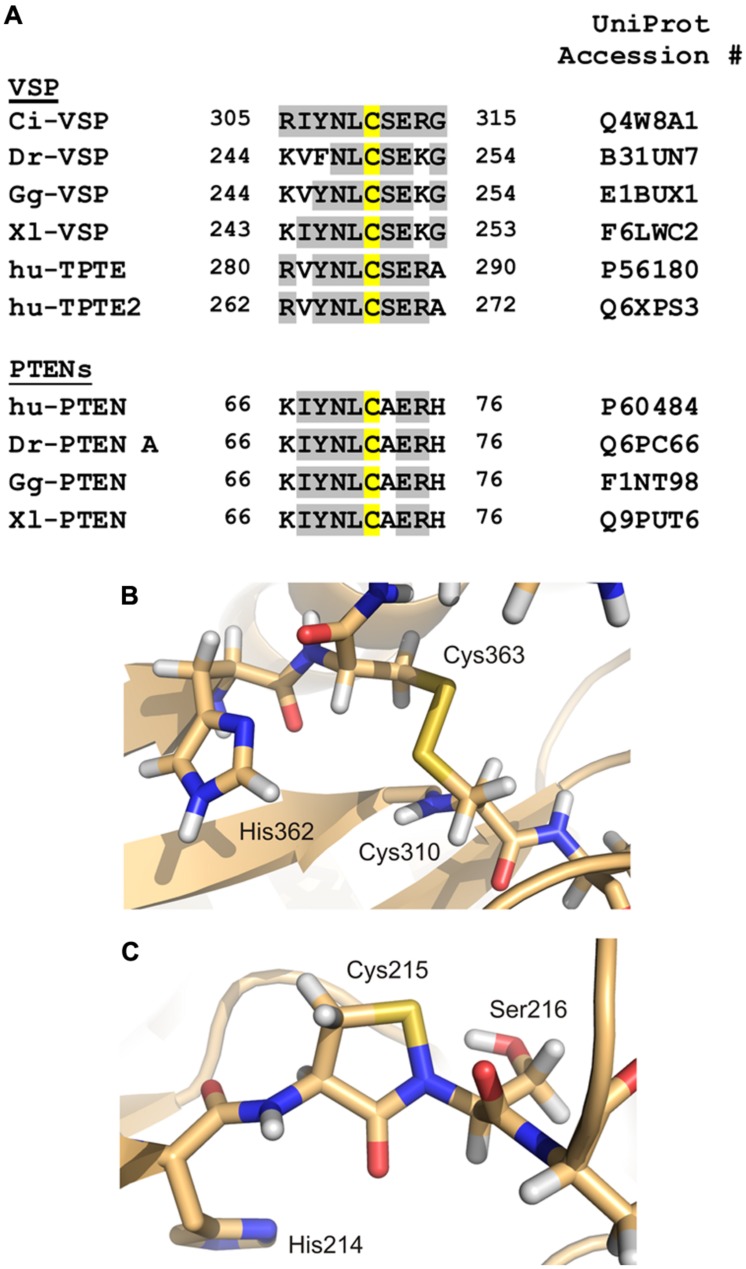
**Redox modification of the catalytically active side chain in cysteine-based PTPs. (A)** The amino acid motif of Ci-VSP, which contains Cys310 as putative interaction partner for the disulfide-bridge with the catalytic Cys363, is aligned with sequences of VSP homologs from various species (Dr, *Danio rerio*; Gg, *Gallus gallus*; Xl, *Xenopus laevis*; hu, human). Additionally, PTEN isoforms from different species are included in this alignment. Amino acid identities to Ci-VSP are colored in gray. Cys310 and the corresponding position in the respective proteins are marked in yellow. **(B)** For Ci-VSP, the disulfide bridge between Cys310 and the catalytic Cys363 is shown as it has been identified in a crystal structure of the cytosolic domain (PDB accession number 3awg; Matsuda et al., 2011). **(C)** In crystal structures of the human PTP1B, the formation of a sulphenyl-amide bond has been observed between the P-loop Ser216 and the catalytically active Cys215 (PDB 1oes; van Montfort et al., 2003).

Another interesting feature about the cysteine in the P-loop is its sensitivity to redox modification. Several studies demonstrated that the presence of redox-reactive agents, such as H_2_O_2_ or NO, reversibly eliminates the enzymatic activity of cysteine-based PTPs (Denu and Tanner, 1998; Xu et al., 2002; Salmeen and Barford, 2005; Tonks, 2005; Ross et al., 2007; Conway et al., 2010; Ostman et al., 2011; Miki and Funato, 2012). For most PTPs, this inhibition is caused by the formation of a disulfide bond between the P-loop cysteine and another cysteine in its immediate environment, as e.g., observed in PTEN (class I; Lee et al., 2002), HCPTP (class II; Chiarugi et al., 2001), and Cdc25B (class III PTP; Buhrmann et al., 2005). In case of PTP1B, the cysteine forms a sulphenyl-amide bond under oxidizing conditions with the nitrogen atom in the backbone of a serine that is located next to the catalytically active side chain (**Figure 5C**; Salmeen et al., 2003; van Montfort et al., 2003). When bound either in the sulphenyl-amide or in the disulfide bond, the sulfhydryl group of the P-loop cysteine is incapable of performing the nucleophilic attack on the phosphate substrate, thus preventing catalysis.

For Ci-VSP, a disulfide bond has been found in one of the crystal structures of the cytosolic domain (**Figure 5B**; Matsuda et al., 2011). This bond was present between the catalytically active residue Cys363 and the neighboring cysteine, Cys310. Accordingly to other PTPs, Ci-VSP does not show phosphatase activity toward PIP substrates in presence of H_2_O_2_ (Matsuda et al., 2011), which demonstrates the inhibiting effect of oxidizing conditions on catalysis.

The alignment of amino acid sequences shows that the cysteine corresponding to position 310 in Ci-VSP is highly conserved in VSPs and also in PTENs (**Figure 5A**). For the latter enzymes, the redox regulation has been well-characterized (Lee et al., 2002; Leslie et al., 2003; Cho et al., 2004; Yu et al., 2005; Kim et al., 2010; Kitagishi and Matsuda, 2013). These facts indicate that the redox regulation of VSPs might follow a similar pattern as described for other PTPs.

#### The invariant arginine

Apart from the P-loop cysteine, the invariant arginine in the active site motif has frequently been found to be critical for the correct binding of the substrate in the active site (**Figure 2B**; Stuckey et al., 1994; Jia et al., 1995; Barford et al., 1998). Furthermore, this residue has been proposed to be responsible for the stabilization of the cysteinyl-phosphate intermediate during the reaction cycle (Zhang et al., 1994c; Pannifer et al., 1998). Based on crystal structure analyses of the soluble phosphatase domain of Ci-VSP, Liu et al. (2012) suggested that this arginine probably marks the position of the phosphate at the substrate that has to be cleaved.

#### Glycine versus alanine and the influence on substrate specificity

A further interesting residue in the P-loop motif is the amino acid two positions more C-terminally from the catalytic cysteine. While PTEN carries an alanine at this position, a glycine is found there in VSPs (**Figure 3A**). PTEN specifically dephosphorylates the 3′-phosphate from PI(3,4,5)P_3_ (Maehama and Dixon, 1998; Maehama et al., 2001; Iwasaki et al., 2008), whereas Ci-VSP shows a broader substrate spectrum by cleaving the 5′-phosphate from PI(3,4,5)P_3_ and PI(4,5)P_2_ (Iwasaki et al., 2008; Halaszovich et al., 2009) and – presumably with less efficiency – the 3′-phosphate from PI(3,4)P_2_ (Sakata et al., 2011; Liu et al., 2012; Sakata and Okamura, 2013). This difference raises the question whether the substrate specificity of VSPs will be changed if the glycine in the active site is substituted against alanine to mimic the substrate binding pocket of PTEN.

In Ci-VSP, the mutation G365A significantly decreases the phosphatase activity toward PI(3,4,5)P_3_ and PI(4,5)P_2_ *in vitro* and *in vivo* (Iwasaki et al., 2008; Liu et al., 2012). However, results about an apparent 3′-phosphatase activity of the G365A mutant are ambivalent. The purified catalytic domain of Ci-VSP which lacks the transmembrane part of the protein shows increased activity toward PI(3,4)P_2_ and PI(3,5)P_2_ *in vitro* in comparison to the wild type (Iwasaki et al., 2008). In contrast to that, the phosphatase activity of the G365A full-length protein was significantly reduced *in vivo* toward PI(3,4)P_2_ compared to the wild type (Liu et al., 2012). These results clearly demonstrate that data obtained by *in vitro* and *in vivo* assays can diverge for VSPs. The reason for this could be that the active site might adopt a different conformation under *in vitro* conditions than *in vivo*, since the structural stabilization of the substrate binding pocket through interactions with the membrane surface or the voltage sensor domain cannot be achieved in these experiments.

Interestingly, the G365A mutant was still capable to deplete PI(4,5)P_2_ *in vivo* (Liu et al., 2012). Thus, the mutation in the P-loop alone is not efficient to convert Ci-VSP into an exclusive 3′-phosphatase like PTEN. Further studies are required to identify the structural determinants for the substrate specificity in VSPs.

#### Specificity regarding protein- or lipid-derived substrates

Last but not least, the amino acid five residues C-terminally from the active site cysteine (**Figure 3A**) also exhibits interesting properties. In PTEN, the G129E mutation eliminates PIP-specific phosphatase activity without affecting the activity toward peptide-derived substrates (Furnari et al., 1997; Myers et al., 1998; Ramaswamy et al., 1999). Gly129 is located at the bottom of the substrate binding pocket. On the basis of the crystal structure of PTEN, it was proposed that the G129E mutation might result in the reduction of the wide access to the active site. In turn, this might prevent the accommodation of PIP substrates, but does not eliminate the capacity to bind phosphoprotein-substrates (Lee et al., 1999).

In case of VSPs, no activity toward peptide-derived substrates has been demonstrated so far. Because of the structural similarity to PTEN, it would be interesting to see if VSPs are also capable to dephosphorylate this kind of substrates in addition to PIP molecules.

### THE WPD- OR DPYY-LOOP

The so-called WPD-loop of class I PTPs is located several amino acid positions N-terminal to the P-loop (**Figure 3**). The name of this region is derived from three amino acids in this motif, which are conserved in several phosphatases, as for example in PTP1B, YopH, and CD45 (**Figure 3A**). The nomenclature of this loop persists for historical reasons, although some members of the PTP family have been discovered that show considerable variations in the WPD-loop sequence.

In case of class II PTPs, the motif which corresponds to the WPD-loop is also highly conserved. Here, this region is termed DPYY-loop, again because of its conserved sequence pattern (**Figure 3A**; Tabernero et al., 1999).

#### The invariant aspartate

The most important residue in the WPD- or the DPYY-loop is the invariant aspartate. Besides the cysteine and arginine conserved in the P-loop, this residue is the third one within the group of most crucial actors in the enzymatic reaction. It mediates the formation of the cysteinyl-phosphate intermediate by acting as general acid during the first step of the catalytic cycle. In the second step, it acts as general base by accepting the proton from an activated water molecule, which enables the reconfiguration of the active site for a new reaction cycle (**Figure 4**; Taddei et al., 1994; Zhang et al., 1994b; Denu et al., 1995a, 1996).

For class I PTPs, mutations of the invariant aspartate reduce or abolish the phosphatase activity (Zhang et al., 1994c; Lohse et al., 1997; Denu and Dixon, 1998). In case of rPTPα, a receptor-like classical PTP, the substitution by glutamate preserves the charge at this position, but changes the steric conditions in such a way that the catalytic activity is decreased at least by two orders of magnitude (Flint et al., 1997; Wu et al., 1997). However, substitution of the aspartate in rPTPα by alanine completely eliminates the catalytic activity toward pTyr (Wu et al., 1997).

Interestingly, it is known from studies on different PTPs that an aspartate-to-alanine mutation in the WPD- or DYPP-loop generates highly efficient substrate-trapping mutants (Garton et al., 1996; Flint et al., 1997; Tonks and Neel, 2001; Blanchetot et al., 2005; Monteleone et al., 2012). Garton et al. (1996) suggested that the mutation enhances the hydrophobic properties of the active site cleft and removes the repulsive electrostatic potential between the aspartate and the phosphate moiety of the substrate. In this way, the substrate-bound conformation could be stabilized (Garton et al., 1996).

In agreement with these results, the corresponding D331A mutation in Ci-VSP drastically slows down the off-motion of the voltage sensor domain after a voltage stimulation (Kohout et al., 2010). This indicates a high energy barrier for the conformational transition of the sensor domain into the resting state, which could be due to the fact that the WPD mutation leads to a trapping of the catalytic domain at its substrate at the membrane surface. Interestingly, the D331A mutation shows a residual phosphatase activity toward PI(3,4,5)P_3_ vesicles *in vitro*, but was completely inactive toward PI(4,5)P_2_ *in vivo* (Kohout et al., 2010). These results imply a drastic slow-down of the catalytic turnover rate, which might arise from substrate trapping also in Ci-VSP.

Since the results obtained from Ci-VSP agree with those from other PTPs, it can be assumed that the aspartate in the WPD-loop of VSPs might play a similar role in catalysis as it does for class I and II PTPs.

#### A glutamate instead of an aspartate with consequence for substrate specificity

Quite recently, an “unusual” PTP has been identified that does not fit into this strict pattern of PTPs with the invariant aspartate in the WPD-loop: the receptor-like class I phosphatase PTPRQ. This enzyme contains a glutamate at the position corresponding to the aspartate in other PTPs. Remarkably, receptor-like PTPs had only been classified as classical PTPs so far. However, PTPRQ has only little activity toward pTyr substrates, but displays specificity for phosphoinositides (Oganesian et al., 2003; Seifert et al., 2003; Park et al., 2013; Yu et al., 2013). Furthermore, in contrast to PIP-specific PTPs like VSPs and PTENs, which show a substrate preference for one position at the inositol ring, PTPRQ dephosphorylates the 3′- and 5′- phosphates of the PIP head group (Oganesian et al., 2003). Notably, if the glutamate in the WPE-loop of PTPRQ is substituted by an aspartate mimicking the sequence pattern of other PTPs, the phosphatase is converted into a pTyr-specific phosphatase with no activity toward PIP substrates (Oganesian et al., 2003).

### THE TI-LOOP IN CLASS I PTPs VERSUS THE V-LOOP IN CLASS II PTPs

The third important loop in the catalytic domain of class I PTPs is the TI-loop. Also here, the name results from a Thr-Ile pair in this region that is conserved in many (including PTEN), but not all PTPs (**Figure 3A**). Remarkably, the overall amino acid sequence of the TI-loop is not as highly conserved as the one of the P- and WPD-loop, which makes it difficult to create a reliable sequence alignment of this region. A structural alignment of different crystallized phosphatase domains on the basis of the P-loop conformation finally enables the identification of the region that corresponds to the TI-loop (**Figures 3B,C**). The motives found by this structural comparison are aligned in **Figure 3A**. This alignment illustrates the high sequence variation in this loop. However, it further shows a notable similarity for class I PTPs: despite the sequence variation, most of these PTPs contain at least one (i.e., PTEN) or even two glutamines (i.e., PTP1B, YopH, CD45) in the TI-loop. Therefore, this region is often also referred to as Q-loop for class I PTPs (Tabernero et al., 2008).

For class II PTPs, little homology of the V-loop to the corresponding TI-loop of class I PTPs can be observed (**Figure 3A**). An additional structural alignment of both regions revealed also no significant similarity, which makes the informational content of the amino acid alignment in **Figure 3A** vague. However, as the TI-loop in class I PTPs, the V-loop in class II PTPs contributes to one of the side walls of the substrate binding pocket (**Figure 3C**). In addition to that, class II PTPs differ in their capability to respond to activators and inhibitors, which is caused by variations in the amino acid sequence of the V-loop regions (Zhang et al., 1998; Madhurantakam et al., 2005). Taken together, the V-loop plays an important role for catalysis in class II PTPs, although it seems to be completely different from the role of the TI-loop in class I PTPs.

#### Glutamine versus threonine versus glutamate in the TI-loop in class I PTPs

**FIGURE 6:**
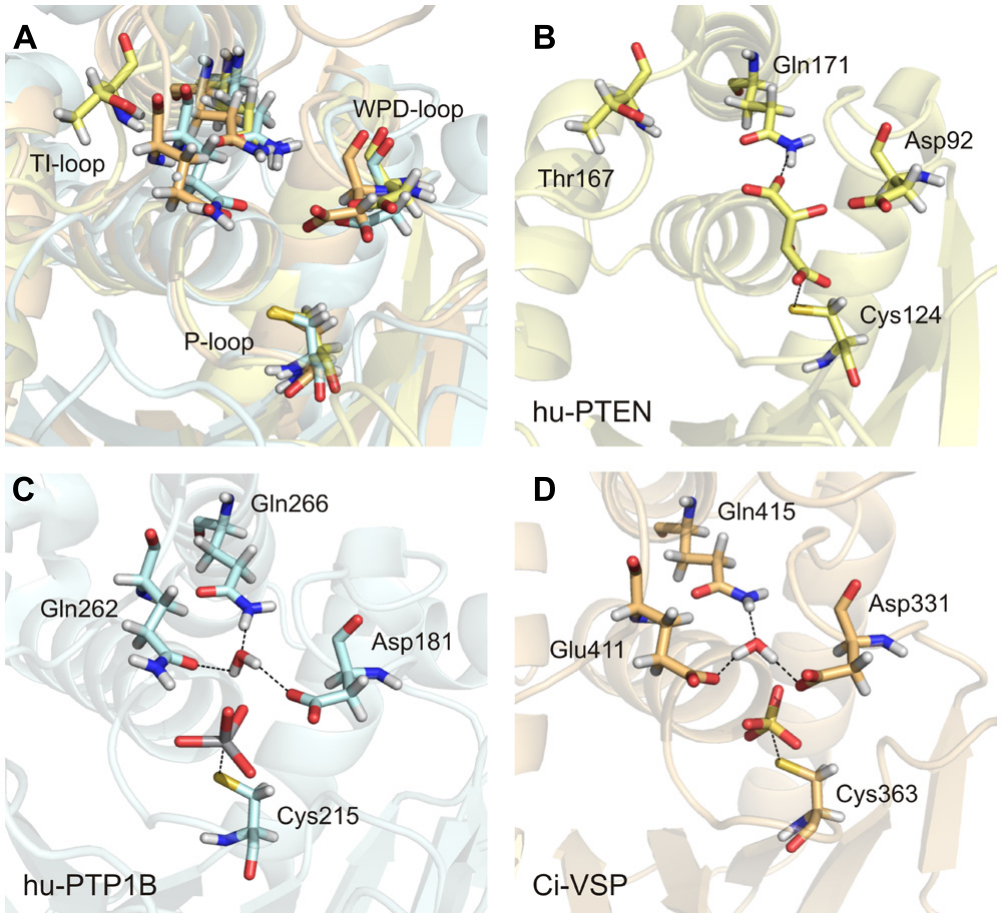
**An activated water molecule is required for the second step in catalysis of class I PTPs. (A)** The substrate binding pocket is structurally aligned for the human PTP1B (in light blue, PDB accession number 3ib0), human PTEN (in yellow, PDB 1d5r), and Ci-VSP [in orange, modeled by MD simulation based on the crystal structure of Matsuda et al. (2011) and Hobiger et al. (2013) PDB 3awe]. The glutamines from the TI-loop of hu-PTP1B and the corresponding amino acids in the other PTPs, the invariant aspartate from the WPD-loop, and the catalytic cysteine from the P-loop are represented as sticks. **(B–D)** The substrate binding pockets are shown individually for the three PTPs with the following ligands bound to the active site, as found in the respective crystal structures: L(+)-tartrate in hu-PTEN, vanadate in hu-PTP1B, and sulfate in Ci-VSP. One water molecule is coordinated adjacently to the aspartate and the ligand by the TI-loop glutamines in hu-PTP1B **(C)** and by corresponding residues in Ci-VSP **(D)**. **(B)** This water molecule is absent in hu-PTEN. Thr167 that corresponds to Gln262 in hu-PTP1B and Glu411 in Ci-VSP is oriented away from the active site. One of the oxygen atoms of the bound tartrate forms a hydrogen bond with the nitrogen of Gln171.

There are many crystal structures of class I PTPs available that contain a compound in the active site that mimics the bound substrate. In these structures, the conserved glutamines of the TI-loop stabilize a water molecule, which is required for the hydrolysis of the cysteinyl-phosphate intermediate during the second step of the enzymatic reaction. For example, in structures of PTP1B with a ligand bound to the active site, the two glutamines Gln262 and Gln266 coordinate the water molecule (**Figure 6C**; Brandao et al., 2010). This configuration has been further proposed to be crucial in class I PTPs for positioning the WPD-loop in an optimal conformation for catalysis (Pannifer et al., 1998; Brandao et al., 2010).

With this knowledge at hand, the question remains: what role does the TI-loop play in VSPs? In these phosphatases, two conserved glutamines exist in the TI-loop, e.g., Gln408 and Gln415 in Ci-VSP (**Figure 3A**). During the catalytic reaction, both residues could putatively act as interaction partners for the coordination of the water molecule. Therefore, we performed a structural alignment of the phosphatase domains of Ci-VSP and PTP1B (**Figure 6**). This comparison reveals that in Ci-VSP, Glu411 instead of Gln408 overlaps with Gln262 in PTP1B (**Figure 6A**). In this configuration, Gln415 of Ci-VSP is positioned in such a way that it corresponds to Gln266 in PTP1B (**Figure 6C,D**). Furthermore, the model of Ci-VSP, which was obtained by MD simulations (Hobiger et al., 2013) predicts the coordination of a water molecule by Glu411 and Gln415 in the immediate environment to the phosphate group of the substrate (**Figure 6D**). The invariant aspartate in the WPD-loop of Ci-VSP, Asp331, additionally participates in this configuration; analogously to the aspartate in the WPD-loop of PTP1B (**Figure 6C,D**).

It should be noted here, that no direct experimental evidence exists so far, whether this configuration is indeed adopted during the catalytic cycle of Ci-VSP. Thus, our proposal just illustrates a hypothetical scenario based on modeling data. However, studies on Glu411 mutants of Ci-VSP support our hypothesis. Recently, Glu411 has been shown to be critical for catalysis in Ci-VSP (Matsuda et al., 2011; Liu et al., 2012). Substitution of Glu411 against hydrophobic residues (Ile, Leu, or Phe) reduced the activity toward PI(3,4,5)P_3_ *in vitro* and almost abolished activity toward PI(4,5)P_2_ *in vivo* (Liu et al., 2012). Crystal structures of the phosphatase domain of Ci-VSP suggested that Glu411 could compete with the substrate for binding to the active site (Matsuda et al., 2011; Liu et al., 2012). Even more so, Liu et al. (2012) proposed that Glu411 may act as molecular switch by changing its position in such a way that the active site opens or closes; a mechanism that was similarly described for Gln262 in PTP1B (Brandao et al., 2010).

Interestingly, in the phosphatase domain of PTEN, which is structurally highly homologous to that one of Ci-VSP, a threonine is located at the position corresponding to Glu411 (**Figure 3A**). Substitution of Glu411 against threonine in Ci-VSP causes a moderate decrease in the phosphatase activity toward PI(3,4,5)P_3_ *in vitro* and PI(4,5)P_2_ *in vivo*, and a strong reduction of its activity toward PI(3,4)P_2_ *in vivo* (Liu et al., 2012). Taken together, these findings suggest that a hydrophobic residue is less well-tolerated at this position for the catalytic activity of Ci-VSP compared to the polar side chain of threonine. This supports our hypothesis that Glu411 might participate in coordinating the water molecule by electrostatic interactions during the second step of catalysis.

But can the threonine in PTEN participate in the coordination of a water molecule? Such a configuration can be studied in the crystal structure of PTEN (Lee et al., 1999). A view into the active site structure of PTEN with a bound tartrate molecule shows that Thr167, which corresponds to Glu411 in Ci-VSP and Gln262 in PTP1B, is oriented away from the substrate binding pocket (**Figure 6B**). Such a configuration would contradict the assumption that Thr167 is involved in the water molecule coordination. However, it should be noted that the tartrate bound in the active site inhibits the catalytic activity of PTEN (Lee et al., 1999). This might be caused by the fact that upon binding of tartrate, Thr167 could be pushed into a position that prevents its participation in catalysis and, consequently, eliminates phosphatase activity. Therefore, it is possible that the crystal structure of PTEN does not represent the catalytically active conformation of the active site. Moreover, mutations T167A and Q171A in PTEN, which would correspond to Q262A and Q266A in PTP1B, and E411A and Q415A in Ci-VSP, reduced phosphatase activity *in vitro* by 60 to 75%, respectively (Lee et al., 1999). These results suggest a functional relevance for both amino acids in the enzymatic reaction of PTEN.

In conclusion, we suggest that the TI-loop in Ci-VSP is directly involved in catalysis since it contains residues that might coordinate the water molecule for the second step in the reaction cycle, namely Glu411 and Gln415. Changing the electrostatic properties by introducing a hydrophobic residue instead of Glu411 eliminates or significantly reduces catalytic activity, whereas the substitution by a polar side chain such as threonine does not abolish enzymatic function, but causes changes in substrate specificity. However, further studies are required to elucidate exactly the interactions in the active site of VSPs.

#### Other charged positions in the TI-loop of class I PTPs

Apart from Glu411 in Ci-VSP, at least one additional charged side chain is located at the N-terminal part of the TI-loop in VSPs. Again, a sequence comparison with other PTPs reveals large variations in this region (**Figure 3A**), which makes it difficult to speculate about the role of these charged residues for VSPs in comparison to the results obtained for PTPs.

In case of Ci-VSP, recent studies suggested that Asp400 in the N-terminal part of the TI-loop interacts with the region that links the phosphatase to the adjacent voltage sensor domain (Liu et al., 2012; Hobiger et al., 2013). Notably, the linker motif of Ci-VSP is assumed to be critical for the coupling between the catalytic and the voltage sensor domain (Murata et al., 2005; Hossain et al., 2008; Villalba-Galea et al., 2009; Kohout et al., 2010; Lacroix et al., 2011; Hobiger et al., 2012). In particular, the interaction between the linker and Asp400 was suggested to be crucial for stabilizing the substrate binding pocket in a voltage-dependent manner (Liu et al., 2012; Hobiger et al., 2013). This process might be one critical step in transferring the conformational change from the voltage sensor to the catalytic domain upon voltage stimulation.

In PTEN, the region that is homologous to the linker of VSPs has been proposed to act as phospholipid binding motif (PBM), because it mediates the targeting of the enzyme to PIP-containing membranes (Campbell et al., 2003; Das et al., 2003; Iijima et al., 2004; Walker et al., 2004; Vazquez and Devreotes, 2006; Redfern et al., 2008). Analogously, this function has also been suggested for the linker in Ci-VSP (Villalba-Galea et al., 2009; Kohout et al., 2010; Hobiger et al., 2012). Deletion of the PBM retains phosphatase activity of PTEN toward water-soluble substrates, but eliminates the activity toward membrane-bound substrates (Iijima et al., 2004; Vazquez et al., 2006). In contrast to that, the purified catalytic domain of Ci-VSP does not show phosphatase activity *in vitro* if the linker is deleted, neither toward membrane-bound substrates nor the soluble PIP head group Ins(1,3,4,5)P_4_ (Kohout et al., 2010).

Although the PBM in PTEN shares structural and functional similarities with the linker in Ci-VSP, no direct interactions between the PBM and regions in the catalytic domain have been described for PTEN up to now. Upon membrane binding of the PBM, conformational changes occur in the catalytic domain leading to the assumption of an allosteric activation mechanism in PTEN (Campbell et al., 2003; McConnachie et al., 2003; Iijima et al., 2004; Vazquez and Devreotes, 2006; Redfern et al., 2008). However, the details of this process are still elusive and the question remains, whether the interaction between the linker and the TI-loop observed for Ci-VSP is an exclusive property of this protein.

## A MODEL FOR THE CATALYTIC MECHANISM IN VSPs

Including all results discussed above, we suggest the following two-step catalytic reaction mechanism for Ci-VSP as representative for all VSPs.

During the first step in catalysis, the binding of the phosphorylated substrate is enabled by defined structural properties of the active site. Here, the phosphate group that will be cleaved from the substrate is positioned by the P-loop Arg369, and a network of hydrogen bonds formed by the backbone atoms of the active site loop. The catalytic Cys363 is oriented in its position by a hydrogen bond with the adjacent Thr370. This configuration stabilizes the sulfur of the cysteine in an anionic thiolate form. All these factors ensure optimal reaction conditions, so that Cys363 acts as nucleophile attacking the phosphate group of the substrate. With the assistance of Asp331 from the WPD-loop, which donates a proton to the substrate leaving group, a cysteinyl-phosphate intermediate is formed, and the dephosphorylated substrate dissociates from the active site pocket.

To return into the resting state, the phosphorus in the reaction intermediate is attacked by an activated water molecule. The activation of the water molecule is achieved by the coordination through Glu411 and Gln415 from the TI-loop. Furthermore, one of the protons of the water molecule is accepted by Asp331. In this way, the phosphate group gets hydrolyzed, so that the initial conformation of the active site is restored to start a new catalytic cycle.

## SUMMARY

Research on VSPs is still an emerging field in membrane biophysics and cell biology. Since the activity of VSPs is switchable by voltage stimulation, these enzymes have been mainly characterized by electrophysiological methods. Taking their structural identity and sequence pattern into account, VSPs can be classified as members of the huge protein family of cysteine-based PTPs. They dephosphorylate specifically PIP-lipids instead of phosphotyrosine substrates. Therefore, and because of the structural properties of their catalytic domain, VSPs belong to the subclass of dual-specific class I PTPs.

The aim of this review is to point out that the information presently available about VSPs and cysteine-based PTPs can be well-integrated into a set of common principles. In particular, we showed that all cysteine-based PTPs including VSPs share a common folding pattern of their catalytic domain. In addition, the two-step reaction mechanisms proposed for class I to III PTPs can also be assumed for VSPs, since all results obtained so far from this latter phosphatase family match well with the results about well-characterized cysteine-based PTPs, such as PTP1B, CD45, VHR, or VH1. However, many of the suggestions made here still need experimental verification. By linking the knowledge about VSPs and PTPs, we want to inspire further research on VSPs to contribute to and to benefit from efforts in the field of the large phosphatase family to which they belong.

## Conflict of Interest Statement

The authors declare that the research was conducted in the absence of any commercial or financial relationships that could be construed as a potential conflict of interest.

## Footnotes

1 A list of all abbreviations used in this text is given in Table S1.

2 A variant of the TPTE family has been initially named as TPIP (TPTE and PTEN homologous inositol lipid phosphatase; Walker et al., 2001), nowadays also called TPTE2. To introduce a more consistent nomenclature, the human isoforms of TPTEs, TPTE2 and TPTE, have also been referred to as hVSP1 and hVSP2, respectively (Halaszovich et al., 2012).
