# Supplementary material for: Voltage sensitive phosphatases: emerging kinship to protein tyrosine phosphatases from structure-function research
Source: Front Pharmacol. 2015 Jan 10;6:20. doi: 10.3389/fphar.2015.00020 (PMC4322731; doi:10.3389/fphar.2015.00020)
Supplement: Supplementary file 1 [file Table_1.PDF]

Frontiers in Pharmacology

Supplementary Material

Voltage sensitive phosphatases: emerging kinship to protein tyrosine phosphatases from structure-function research

Kirstin Hobiger[1]*, Thomas Friedrich[2]
[1]Philipps-Universität Marburg, Institute of Physiology and Pathophysiology, Dept. Neurophysiology, Deutschhausstr. 1-2, 35037 Marburg, Germany
[2]Technische Universität Berlin, Institute of Chemistry, Max-Volmer-Laboratory of Biophysical Chemistry, Sekr. PC14, Str. des 17. Juni 135, 10623 Berlin, Germany
*** Correspondence:** Kirstin Hobiger, Philipps-Universität Marburg, Institute for Physiology and Pathophysiology, Dept. Neurophysiology, Deutschhausstr. 1-2, 35037 Marburg, Germany, kirstin.hobiger@staff.uni-marburg.de

Supplementary Table 1. List of abbreviations used in the main text.

**Abbreviation**	**Explanation**
ACP	Acid phosphatase
ACP1	Gene 1 coding for acid phosphatase
BHPTP	Bovine heart PTP
CD	Cluster of differentiation
Cdc	Cell division control protein
Cdk	Cyclin-dependent kinase
Ci-VSP	Voltage sensitive phosphatase from *Ciona intestinalis*
c-Jun	Human protein encoded by the *JUN* gene
D1/2	Phosphatase domain 1/2 in receptor-like class I PTPs
DPYY-loop	One of the loops surrounding the active site of class II PTPs (name derived from conserved amino acids in this loop)
DUSP	Dual-specific phosphatase
EMP2A	Gene 2A coding for the enamel matrix protein
Erk	Extracellular-signaling-regulated kinase
HCPTPA/B	Human red cell PTP form A/B

Abbreviation	Explanation
hVSP1/2	Human voltage sensitive phosphatase form 1/2
IF1/2	Isoform 1/2 (used for splice forms 1/2)
INPP4	Inositol-4-phosphatase
$Ins(1,3,4,5)P_4$	Inositol-(1,3,4,5)-tetraphosphate
Jnk	c-Jun N-terminal kinase
JUN	Oncogene homologous to the avian sarcoma virus 17 (name "*JUN*" derived from the Japanese word "ju-nana", meaning number 17)
LMW-PTP	Low molecular weight phosphatase
LMW-PTP-C	Low molecular weight phosphatase form C
MAP	Mitogen activated protein
MAPK	Mitogen activated protein kinase
MD simulation	Molecular dynamics simulation
MKP	MAPK phosphatase
MTM	Myotubularin
PBM	Phospholipid binding motif
PDB	Protein data bank
PI	Phosphoinositide
PI3K	Phosphoinositide-3 kinase
PIP	Phosphoinositide phosphate
PIR1	Phosphatase interacting with RNA-ribonucleoprotein complex 1
P-loop	PTP recognition loop
PRL	Phosphatase of regenerating liver
pSer	Phosphoserine
PTEN	Phosphatase and tensin homolog
pThr	Phosphothreonine
PTP	Phosphotyrosine phosphatase
PTP1B	Phosphotyrosine phosphatase 1B
PTPRQ	Phosphotyrosine phosphatase receptor Q
pTyr	Phosphotyrosine

Abbreviation	Explanation
rPTP	Receptor-like phosphotyrosine phosphatase
SSH	Phosphoserine specific slingshot
Stat	Signal transducer and activator of transcription
SV3	Splice variant 3
TI-loop	One of the loops surrounding the active site of class I PTPs (name derived from conserved amino acids in this loop)
TPIP	TPTE and PTEN homologous inositol lipid phosphatase
TPTE	Transmembrane phosphatase with tensin homology
VH1	Phosphatase encoded by the vaccinia virus gene *H1*
VHR	Phosphatase related to VH1
V-loop	Variable loop; one of the loops surrounding the active site of class II PTPs (name derived from the variability in amino acid sequences among class II PTPs)
VSP	Voltage sensitive phosphatase
WPD-loop	One of the loops surrounding the active site of class I PTPs (name derived from conserved amino acids in this loop)
YopH	*Yersinia* outer protein H (PTP from *Yersinia pestis*)
